# Liquid chromatography as candidate reference method for the determination of vitamins A and E in human serum

**DOI:** 10.1002/jcla.23528

**Published:** 2020-10-08

**Authors:** Qingqing Pan, Min Shen, Ting Yu, Xiaodong Yang, Quanle Li, Beibei Zhao, Jihua Zou, Man Zhang

**Affiliations:** ^1^ Reference Laboratory MedicalSystem Biotechnology Co., Ltd. Ningbo Ningbo China; ^2^ Department of Clinical Laboratory Beijing Shijitan Hospital Beijing China; ^3^ Division of In Vitro Diagnostics for Non‐infectious diseases National Institutes for Food and Drug Control Beijing China; ^4^ Independent Clinical Laboratory Guangzhou Kingmed Diagnostics Group Co., Ltd. Guangzhou China

**Keywords:** candidate reference method, HPLC, vitamins A and E

## Abstract

**Background:**

Owing to the increasing interest in public health research of antioxidant micronutrients and the inaccuracy of routine serum concentrations of the fat‐soluble vitamins A (retinol) and E (DL‐α‐tocopherol) measurements, we developed a reliable, highly sensitive, robust and rapid method for the quantification of two clinically important lipophilic antioxidants in serum using a reverse‐phase HPLC/DAD method.

**Method:**

Sample preparation and analytical conditions that would affect extraction efficiency and quantitative results of vitamins A and E were investigated and optimized. Vitamins A and E were extracted from serum via liquid‐liquid extraction (LLE). After adequate sample preparation, the samples were injected directly into the HPLC system with diode‐array detector (DAD). Chromatographic separation was completed in 7 minutes for vitamins A and E. With vitamin A acetate and vitamin E acetate as internal standards, the method was applied to the measurement of vitamins A and E in human serum.

**Results:**

We evaluated method linearity, accuracy (recovery rate and trueness), precision, carryover, limit of quantitation and limit of detection, and measurement uncertainty. The method was evaluated for trueness using NIST Standard Reference Material SRM 968f. The serum concentration of the studied compounds had a good linear relationship in the range of 0.05 ~ 3.0 μg/mL concentration (*r* ＝ 0.9998), with 0.0077 μg/mL detection limit and 0.025 μg/mL quantitative limit for vitamin A, respectively, and 1.0 ~ 60.0 μg/mL concentration (*r* ＝ 0.9999), with 0.40 μg/mL detection limit and 0.50 μg/mL quantitative limit for vitamin E, respectively. The intra‐ and inter‐assay coefficients of variation were calculated by using three concentrations (1, 2, and 3) of the studied compounds in human serum samples. Intra‐assay and inter‐assay precision were 1.23%‐4.97% and 0.97%‐3.79% for vitamin A, respectively, and 0.64%‐4.07% and 0.81%‐5.96% for vitamin E, respectively. The average recovery rates were 100.98% for vitamin A, and 99.21% for vitamin E, respectively. The carryover rate of vitamins A and E was below 1%. As for the evaluation of accuracy, the biases were <± 5% by comparing with NIST standard reference material SRM 968f.

**Conclusion:**

The method is a simple sample treatment procedure for the determination of fat‐soluble vitamins A and E in human serum with high sensitivity and specificity. The proposed method could be recommended as a candidate reference method for the determination of serum concentrations of the fat‐soluble vitamins A and E in human serum.

## Graphical abstract

A simple sample treatment procedure for the determination of fat‐soluble vitamins A and E in human serum with high sensitivity and specificity using a reverse‐phase HPLC/DAD method was developed. Method linearity, accuracy (recovery rate and trueness), precision, carryover, limit of quantitation and limit of detection, and measurement uncertainty were evaluated. The proposed method could be recommended as a candidate reference method for the determination of serum concentration of the fat‐soluble vitamins A and E in human serum.

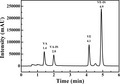

## 1 INTRODUCTION

Oxidative stress, an imbalance between oxidants and antioxidants in favor of the impaired antioxidants potentially leading to damage, is believed to be implicated in the etiopathology of a number of diseases including cholestatic liver disease, exocrine pancreatic insufficiency, cancer, atherosclerosis, neurodegenerative disorders, cardiovascular disease, and other conditions.^1^, ^2^, ^3^ However, interest has grown on the possibility that intracellular and extracellular antioxidants may reduce the risk of oxidative modification. Among fat‐soluble vitamins, vitamins A and E are major components of the antioxidant system in humans, protecting cell membranes against peroxidation,^4^, ^5^, ^6^ maintaining organism metabolism and physiological functions. In general, retinol and DL‐α‐tocopherol are considered to be the most effective biologically active forms of vitamins A and E. In clinical, determination of vitamins A and E is determination of retinol and DL‐α‐tocopherol in human serum concentration. Vitamin A plays a significant role in a wide range of physiological processes in the human body, including maintaining normal growth and reproduction, embryonic development, vision, immunity response, and neurogenesis.^7^, ^8^, ^9^ Vitamin E has the protective effect on other biological antioxidants that are associated with certain cancers,^10^ chronic diseases,^11^, ^12^ diabetic complications,^13^ neurological, and reproductive processes.^9^ Both excess and deficiency of vitamins A and E can cause adverse effects, such as nyctalopia, cardiovascular disease,^14^ abnormal fetal development, disruption to energy balance regulation,^15^ hemolytic anemia in premature, newborn, and infants progressive neurological degeneration, and so on. In view of their clinical importance, determination of vitamins A and E in human serum concentration becomes necessary to evaluate the blood antioxidant status.

A variety of methods have been reported to determine the concentration of vitamins A and E in plasma and serum. Thin‐layer chromatography methods,^16^ paper chromatography,^17^ gas chromatography (GC),^18^ capillary electrophoresis (CE)^19^, ^20^ and high‐performance liquid chromatography (HPLC) coupled with the ultraviolet detector (UVD),^21^ fluorescence detector (FLD),^22^ electrochemical detector (ECD),^23^, ^24^ or mass spectrometer (MS)^25^ are more traditional approaches and are widely used in routine clinical measurements. However, the results obtained with different methods are often not comparable because of inter‐method and inter‐laboratory variability. In addition, extremely low level of vitamin A in biological samples needs a large quantity of serum and do not allow for accurate quantification and high sensitivity in a single chromatographic run.^26^, ^27^

The inadequate accuracy of vitamins A and E measurements hampers the interpretation of data in public health research. In order to improve the accuracy of the routine methods in laboratory medicine, establishing a reference measurement system for value‐assigning NIST standard reference material (SRM) and external quality assessment of these vitamins is the best way to help verifying standardization effectiveness. According to our knowledge concerning the field, no reference measurement methods/procedures (RMPs) for quantitation of vitamins A and E in human serum currently have been reviewed and accepted by the Joint Committee for Tractability in Laboratory Medicine (JCTLM).

Based on previous reported work, our developed method is simple, rapid, accurate, reliable, sensitive, selective, and cost‐effective HPLC method with diode‐array detector (DAD), for the simultaneous determination of vitamins A and E in human serum after LLE. Vitamin A acetate and vitamin E acetate were used as internal standards (IS) to improve the accuracy and precision of the method. The method was also validated strictly and comprehensively, and applied to the determination of vitamins A and E in human serum.

## 2 EXPERIMENTAL

### 2.1 Materials and reagents

Analytical standards of vitamin A, vitamin E and the internal standards of vitamin A acetate, vitamin E acetate were obtained from Sigma (Sigma‐Aldrich Co. LLC.). The Standard Reference Material (SRM 968f) was purchased from National Institute of Standards and Technology (NIST). 2,6‐bis (1,1‐dimethylethyl)‐4‐methylphenol (butylated hydroxyl toluene, BHT) was obtained from Sigma (Sigma‐Aldrich Co. LLC.). All used solvents including methanol, ethanol, and hexane (chromatography grade) were purchased from Thermo (Thermo Fisher Scientific Inc). A water purification system (Millipore) was used to provide Milli‐Q water of ultra‐pure quality (18.2 MΩ). Human serum samples were collected from different healthy volunteers.

### 2.2 Chromatographic conditions

High‐performance liquid chromatography analysis was performed on a SHIMADZU LC‐30A system equipped with diode‐array detector. Samples were analyzed using an ODS HYPERSIL C_18_ analytical column (3.0 µm, 100 mm × 2.1 mm) connected with a 2.1 mm × 20 mm guard column at a temperature of 30°C, and the flow rate was kept constantly at 0.5 mL/min. The mobile phase employed a gradient elution using the following constituents: Mobile phase A was water; mobile phase B was methanol. The gradient began with 93% methanol/water for 1 minutes, followed ramping to 100% methanol in 0.01 minutes. Then, 100% methanol was maintained for 2.5 minutes, then returned to initial conditions and equilibrated for 3.5 minutes. The autosampler temperature was 4°C, and the injection volume was 20 μL. The DAD detector was adjusted at 325 nm for vitamin A and vitamin A acetate, 291 nm for vitamin E and vitamin E acetate. The amount of individual vitamins was quantified from the corresponding peak area ratio of vitamin/internal standard (vitamin A/vitamin A acetate; vitamin E/vitamin E acetate) using LabSolutions software.

### 2.3 Calibration preparation and control materials

Volumetric steps were gravimetrically controlled, where indicated, using a model of XS205 DualRange electronic balance (Mettler Toledo) which has a readability of 0.01 mg. All solutions of standards were made using ethanol containing BHT (0.2%, w/v), which is as the antioxidant. Master stock solutions of standards (vitamin A and vitamin E) and internal standards (vitamin A acetate and vitamin E acetate) were gravimetrically individually prepared by dissolving solid material in ethanol containing BHT (0.2%, w/v) to achieve a concentration of approximately 1 mg/mL, 3 mg/mL, 2 mg/mL, and 2 mg/mL, respectively. Six working standard solutions were gravimetrically prepared by combining different amounts from the master stock solutions by dilution with ethanol containing BHT (0.2%, w/v) to achieve a targeted range of vitamin A of 0.05 ~ 3.0 μg/mL and vitamin E of 1.0 ~ 60.0 μg/mL, respectively. Working internal standard solutions (ISTD) were made by gravimetrically combining different amounts from the master stock solutions to achieve concentrations of approximately 0.25 and 25 µg/mL, for vitamin A acetate and vitamin E acetate, respectively. All solutions were stored in amber glass vials at −20°C until analysis.

We prepared all internal quality control (IQC) materials using pooled human serum from healthy volunteers. All units were screened and spiked with either vitamin A or vitamin E, or both, as needed, to achieve desired concentrations, and all specimens were stored at −70°C when not in use.

### 2.4 Sample preparation procedure

Volumetric steps were gravimetrically controlled where indicated. 100 μL serum/working standard solutions was pipetted into a well‐capped 2 mL amber polypropylene tube and accurately weighted. 200 μL of the internal standards was gravimetrically added, and the solution was vortex‐mixed for 2 minutes. After An aliquot (800 μL) of the n‐hexane containing BHT (1 mg/mL) was added, the content mixed vigorously on a vortex mixer for 5 minutes and centrifuged (8000× *g*, 5 minutes, 4°C). The upper hexane layer was transferred into 5 mL amber glass tubes. To maximize extraction efficiency, the LLE step was repeated and the combined organic layers were dried under nitrogen at room temperature. The dried residue was dissolved in 100 μL of 95% methanol/water followed by 2 minutes vortex and centrifugation at 13 000× g for 5 minutes at 4°C. Then, 20 μL of this extracted serum was injected into HPLC for analysis.

The calibration solution was validated prior to use by testing with Standard Reference Material (SRM 968f) (NIST), and we treated SRM 968f using the same procedures to complete the method validation. A standard curve was prepared for each run by extracting and analyzing two aliquots of the calibration solution.

## 3 RESULT AND DISCUSSION

### 3.1 Optimization of high‐performance liquid chromatographic conditions

**FIGURE 1:**
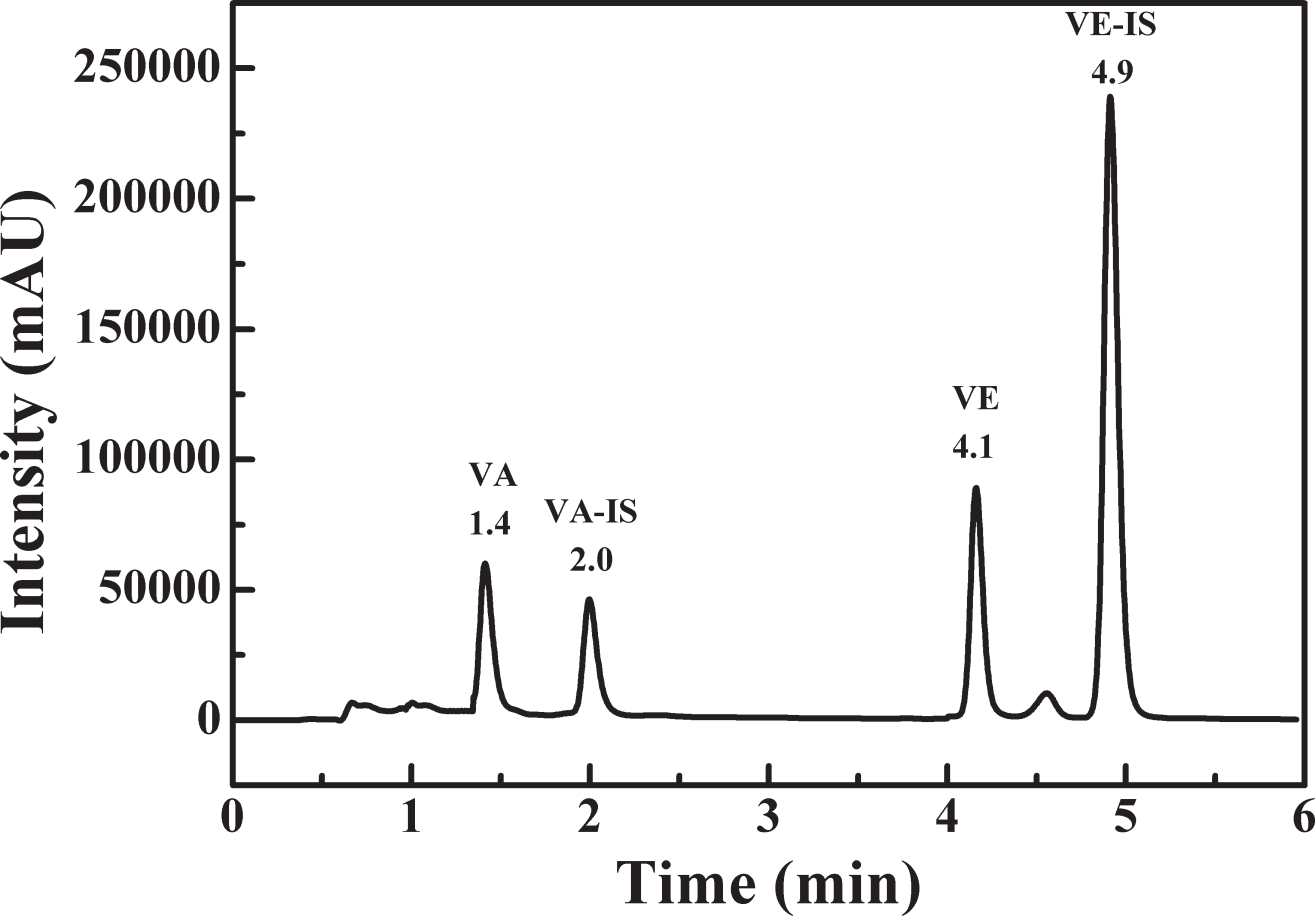
Chromatogram of the mixed standard solutions including vitamin A (3.0 µg/mL), vitamin A acetate (2.0 µg/mL), vitamin E (20.0 µg/mL), and vitamin E acetate (100.0 µg/mL)

**FIGURE 2:**
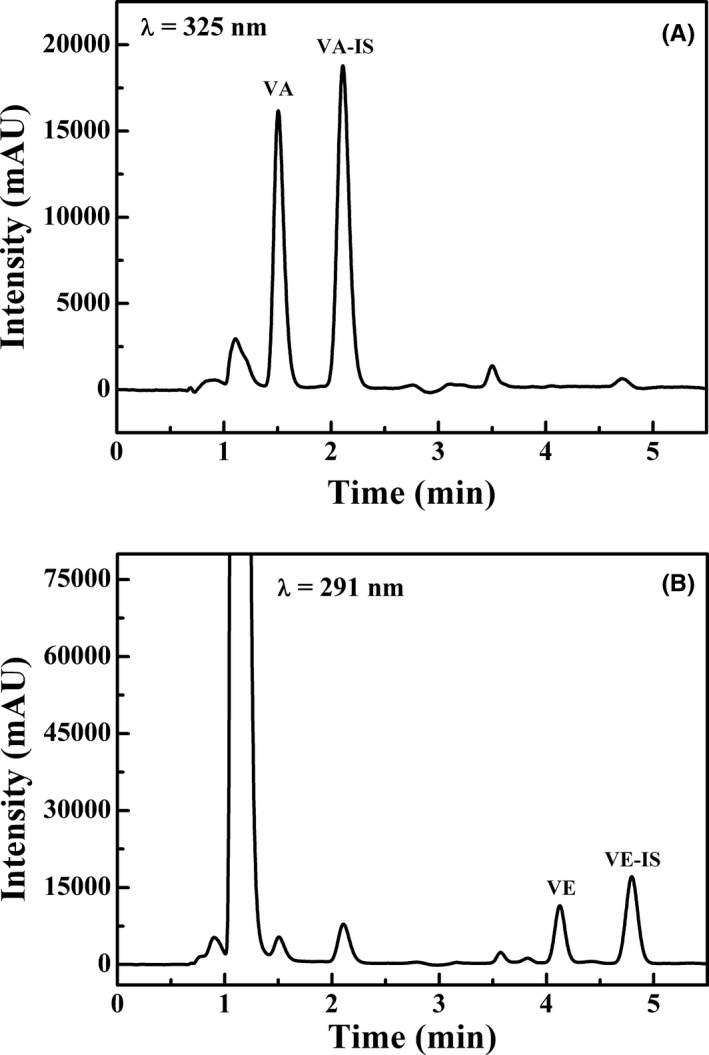
Chromatogram of human serum, (A) vitamin A together with vitamin A acetate was monitored at 325 nm, and (B) vitamin E together with vitamin E acetate was monitored at 291 nm

Analytes were separated on a reversed‐phase column using a gradient system of methanol and water. The mobile phase was optimized in order to obtain the best separation of the analytes in the shortest time. Vitamin A, vitamin E, and internal standards as well as pooled serum samples were used for studying the mobile phase composition. Several elutions (mixtures of organic solvents such as acetonitrile, methanol, ethanol, and formic acid) and several gradients were assessed. And other experimental parameters such as analytical column, detector wavelength, flow rate, column oven temperature, injection volume, and internal standard selection were also optimized. The best results were obtained for the conditions that described in “Chromatographic conditions.” The criteria were resolution, stability of the absorbance, and analysis duration. According to our results, we can conclude that the presented method is highly robust. Representative chromatograms of the HPLC analysis of mixed standard solution and human serum are shown in Figures 1 and 2. Vitamin A, vitamin A acetate, vitamin E, and vitamin E acetate could be separated absolutely, and the retention time of analytes was 1.4, 2.0, 4.1, and 4.9 minutes, respectively. The entire procedure took 7 minutes which is less than half the time required in most previously published protocols^28^, ^29^, ^30^ and separated absolutely. Peak identification was achieved by comparison with the retention time and the spectrum of authentic standards.

### 3.2 Optimization of sample extraction

Sample preparation is essential for accurate analysis. Both pre‐treatments (protein precipitation and liquid‐liquid extraction) were studied. Several protein precipitants (methanol, acetonitrile, ethanol, 2‐propanol, zinc sulfate solution, and strong acid) and different extractants (dichloromethane, diethyl ether, n‐hexane, and ethyl acetate) were tested. Eventually, LLE was selected to extract the target vitamins in this study. And the results showed that the highest extraction efficiency and fewer impurities were obtained by use of n‐hexane. In addition, it well dissolves the fat‐soluble vitamins and does not mix with the water fraction of the blood serum matrix. Therefore, n‐hexane was selected as the extractant for the study. Extraction efficiencies for different volumes of n‐hexane and the effect of vortex time were compared. It was found that the 800 μL n‐hexane could achieve the highest extraction recovery with absolute efficiency ≥75% and the minimum time of mixing of a blood serum sample with the extractant is 5 minutes. To overcome the serum lipoprotein‐binding of the analytes for solvent extraction,^31^ ethanol contained (vitamin A acetate and vitamin E acetate) was added to the samples for protein denaturant before the addition of n‐hexane.

It is very important that extracts were to be dried under nitrogen at room temperature as quickly as possible. Since, both vitamins A and E are light and heat sensitive, also not stable in n‐hexane and ethanol. Hence, it is necessary that the antioxidant (BHT) was used to stabilize the standard solutions or residual extract from serum and the process of sample preparation and handling was carried out in dim light and at room temperature. The concentration of BTH in extraction solvent was optimized in the concentration range of 1‐5 mg/mL, as higher concentration of BHT resulted in large interfering peak that suppressed the analytes peaks in chromatogram.

### 3.3 Method validation

The developed method was validated strictly and comprehensively by evaluating the linearity, accuracy (recovery rate and trueness), precision, carryover, limit of quantitation and limit of detection, and measurement uncertainty. The laboratory results showed that the method was accurate and fully validated for the simultaneous determination of vitamins A and E in serum.

**FIGURE 3:**
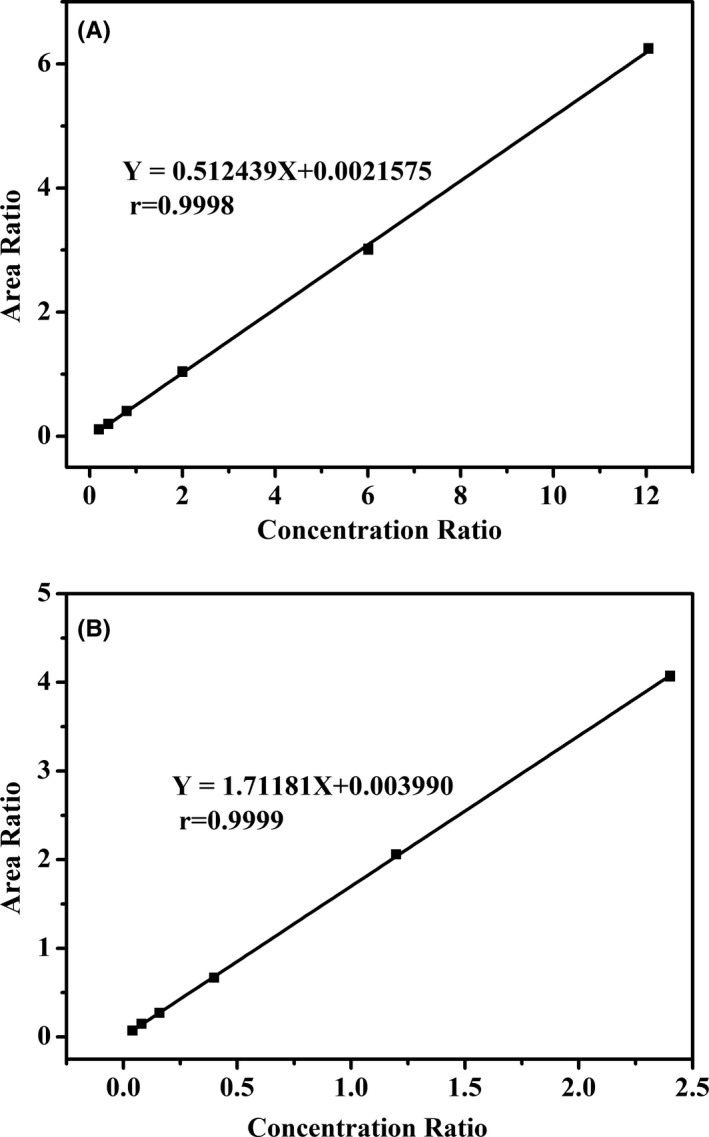
Representative calibration curve for vitamin A (A) and vitamin E (B), all correlation coefficient values were greater than 0.99

The calibration curves of standard solutions showed good linearity with high correlation coefficients in the range of 0.05 ~ 3.0 μg/mL and 1.0 ~ 60.0 μg/mL for vitamins A and E, respectively. Regression equation and correlation coefficient (*r*) calculated from the calibration curves of standard solutions for vitamin A were y = 0.512439x + 0.002158, *r* = 0.9998 and for vitamin E were y = 1.71181x + 0.003990, *r* = 0.9999 (Figure 3). The target for lower limit of quantification of each analyte was based on clinical need and defined as the lowest tested concentration at which the precision was within <20% and analytical recovery was within 100% ± 20%. The confirmed lower limit of quantification varied depending on the analytes but was between 0.025 and 0.5 μg/mL for vitamins A and E, respectively.

Accuracy of the method was determined on the basis of recovery rates with use of serum samples spiked with three different concentration levels of the analytes, and the unspiked serum samples and spiked samples were then analyzed by use of the method. Recovery values were calculated by comparing the amount of vitamins A and E found in the samples to the amount added. As shown in Table 1, the mean recovery of vitamin A was 100.65% and that of vitamin E was 99.67%. Furthermore, the trueness of the proposed method was demonstrated by analyzing the NIST‐certified reference materials. Each level of the material was measured 3 times, and the measurement results were shown in Table 2. The obtained results were all in good agreement with the certified values.

**TABLE 1:** Recoveries of analysis of vitamins A and E (n = 5)

Analyte	Background (µg/mL)	Spiked (µg/mL)	Found (µg/mL)	Recovery (%)	CV (%)	Mean recovery (%)
Vitamin A	0.4099	0.1002	0.5100	99.98	4.02	100.98
		0.5009	0.9170	100.68	2.15	
		1.5028	1.9560	102.26	2.99	
Vitamin E	3.1049	1.9994	5.2000	101.87	1.49	99.21
		9.9971	12.8280	97.91	2.12	
		29.9912	32.3850	97.85	1.06	

**TABLE 2:** Results of analysis of NIST standard reference material 968f (μg/mL)

	Target value^a^	Observed values (n = 3)	
		Mean	Bias (%)
Level I			
Vitamin A	0.327 ± 0.013	0.336	+2.75
Vitamin E	5.15 ± 0.21	4.99	−3.11
Level II			
Vitamin A	0.658 ± 0.028	0.670	+1.82
Vitamin E	11.85 ± 0.73	11.54	−2.62

^a^ Target value certified by NIST.

To test the precision of this method, internal quality control (IQC) materials of serum samples including low‐, medium‐, and high‐concentration serum samples were chosen. Intra‐assay and inter‐assay coefficient of variations (CVs) and total coefficient of variation were determined in terms of repeatability and quantified by the CV of the replicate measurements. As presented in Table 3, the intra‐assay coefficients of variation (CV) of low, middle, and high levels for vitamin A were 4.13%‐4.97%, 1.23%‐2.04%, and 1.24%‐2.16%, and for vitamin E were 1.62%‐4.07%, 1.11%‐2.32%, and 0.64%‐3.14%, respectively (n = 5), whereas the inter‐assay coefficients of variation were 0.97%, 3.79%, and 2.57% for vitamin A and 5.96%, 2.62%, and 0.81% for vitamin E, respectively. The data indicated that the assay method showed good repeatability.

**TABLE 3:** Precision of liquid chromatography analysis of vitamins A and E in serum

Measurand	Specimen	Run	Mean (μg/mL)	Intra CV (%)	Total Mean (μg/mL)	Inter CV (%)	Total CV (%)
Vitamin A	Low‐level	1	0.10	4.13	0.10		4.53
		2	0.10	4.97		0.97	
		3	0.10	4.69			
	Middle‐level	1	0.60	1.23	0.59		3.51
		2	0.60	2.04		3.79	
		3	0.56	1.40			
	High‐level	1	1.15	1.24	1.12		2.78
		2	1.10	2.16		2.57	
		3	1.11	2.03			
Vitamin E	Low‐level	1	2.09	2.46	2.19		5.66
		2	2.13	4.07		5.96	
		3	2.34	1.62			
	Middle‐level	1	9.58	1.86	9.30		2.81
		2	9.14	1.11		2.62	
		3	9.19	2.32			
	High‐level	1	20.98	0.64	20.86		2.04
		2	20.67	1.29		0.81	
		3	20.94	3.14			

To evaluate carryover, samples with high and low concentration were prepared at the upper and lower limit of analytical measurement range and the injection sequence of low‐low‐low‐hi‐hi‐low‐hi‐hi‐low‐low‐low‐low‐hi‐hi‐low‐hi‐hi‐low was performed in triplicate with 3 independent extractions. The mean difference of <20% and <3 SD between low‐low and hi‐low indicated no significant carryover.^32^ SD was calculated based on low control‐1 values. The data indicated that no significant carryover was observed for vitamins A and E.

### 3.4 Measurement uncertainty

The measurement uncertainty of the proposed method is strictly evaluated according to the evaluation procedure of measurement uncertainty which were established on Guide to the Expression of Uncertainty in Measurement (GUM, 1999). When evaluating the calibration and measurement capability (CMC), the different mass concentrations of samples were detected, and their measurement uncertainty was evaluated. With the mole concentration from low to high, the uncertainty of measurement was descending. According to the China National Accreditation Service for Conformity Assessment standard CNAS‐GL 37 “Guidance on Expression of Calibration and Measurement Capability (CMC),” the range of values could be used to represent the calibration and measurement capability and the results were shown in Table 4.

**TABLE 4:** The calibration and measurement capability in our laboratory

Analyte	Measurement range	Expanded uncertainty (CMC) (k = 2)
Vitamin A	(0.10‐1.12) μg/mL	*U*_rel_ = 6.1%‐4.0%
Vitamin E	(2.19‐20.86)μg/mL	*U*_rel_ = 2.7%‐2.0%

## 4 CONCLUSION

In the present study, we developed a reliable, robust, and highly accurate method for the quantification of vitamins A (retinol) and E (DL‐α‐tocopherol) in serum using a reverse‐phase HPLC/UV isocratic method that featured good sensitivity despite using a relatively small amount of serum. The good quality of accuracy and precision values in validation samples confirmed that the proposed method can be applied for the assessment of oxidative stress by monitoring the concentration of vitamins A and E in serum/plasma of healthy volunteers and patients with diabetes and cardiovascular diseases in clinical studies, also could be recommended as a candidate reference measurement procedure in clinical laboratory.
